# Study on the effects of different melting processes on the quality and oxidative stability of milk powder oil raw materials

**DOI:** 10.1016/j.fochx.2025.102888

**Published:** 2025-08-06

**Authors:** Wen Tu, Longyu Wan, Jiaxin Zhang, Huabing Wang, Yadong Huang, Xu Wang, Jian He, Wei Zhang, Qianyu Zhao, Feng Zhao, Yujun Jiang

**Affiliations:** a Key Laboratory of Dairy Science, Ministry of Education, College of Food Science, Northeast Agricultural University, Harbin 150030, China; b Key Laboratory of Infant Formula Food, State Administration for Market Regulation, Harbin 150030, China; c National Technology Innovation Center for Dairy, Huhehaote 010000, China; d Inner Mongolia Yili Industrial Group Co., Ltd., Huhehaote 010110, China

**Keywords:** Melting processes, Oil, Antioxidant capacity, Oxidative stability, Volatile flavor substance

## Abstract

Infant formula milk powder processing technology is complex; the melting treatment of milk powder oil raw materials is also crucial to its quality. It was experimentally concluded that oil needs 29 h, 24 h, 20 h, and 18 h to be completely oiled at 50 °C, 60 °C, 70 °C, and 80 °C, respectively. To investigate the effects of these four processes on oil quality, oxidative stability, and flavor. The acid value of the oil increased by only 3.3 % after 29 h of treatment at 50 °C. Under these conditions, the treated oil showed the lowest free radical peak intensity and longest oxidation induction period. However, oil deterioration was higher at 70 °C for 20 h and 80 °C for 18 h. These results demonstrate that the oil undergoes minimal deterioration at lower temperatures, exhibiting negligible differences from its initial state.

## Highlights

• Melting of oil ingredients at lower temperatures facilitates their quality retention.
• Less loss of antioxidants of oils at low temperatures for long periods of time.
• Oils oxidative stability is worse at high temperatures for short periods of time.

## 1 Introduction

Infant formula serves as a breast milk substitute(Luo et al., 2023). With most commercial formulations deriving their primary protein and fatty acid content from bovine or caprine milk sources(Tang et al., 2024). Currently, different methods are used in infant formulae to adjust the fatty acid composition of the formula(Yang et al., 2024). The main types of lipids commonly used in infant formulas are 1,3-dioleic acid-2-palmitic acid triglycerides(OPO), soya bean oil, etc. The heterogeneous melting points of mixed oil raw materials often result in premature solidification prior to processing. To ensure homogeneity in the final infant formula product, these oils must first be completely liquefied before mixing. Consequently, the oxidative stability and nutritional quality of the oils during the melting phase are critical determinants of the final product's characteristics.

Prolonged heating of milk powder lipid ingredients during the melting stage generates free fatty acids and other degradation products, significantly increasing the acid value (AV) and carbonyl value (GCV) indices. Concurrently, elevated temperatures induce depletion of bioactive compounds, consequently diminishing the oil's antioxidant capacity. Olesea Roman et al. found that hydroperoxides, conjugated dienes(CD) and aldehydes all increased after heat treatment of sunflower oil at different temperatures(Roman et al., 2013). Asadi et al. found that antioxidants extracted reduced CD, AV and GCV content of vegetable oils processed at high temperatures(Asadi & Farahmandfar, 2020). Peroxides produced by lipid oxidation in oil raw materials will not only endanger the health of infants. In addition, secondary oxidation products such as aldehydes, ketones have an impact on the flavor of both the raw oil and the finished milk powder. Edwald Lee et al. found that the content of pentane, hexanal, heptanal, and 1-pentanol in roasted sesame oil increased with increasing storage time(Lee & Choe, 2012). Changes in the oxidative stability of oils during heating have been widely studied. However, changes in oil quality, oxidation products and flavor substances during the pre-treatment stage of oil melting have not been reported. This experiment investigates the changes in quality, oxidative stability and flavor of infant formula oil raw materials under different melting processes, which is important for improving the quality of infant formula from the aspect of oil raw materials.

## 2 Materials and methods

### 2.1 Experimental materials and reagents

Edible Vegetable Blending Oil (Bonjelordes Oils & Fats Technology Co., Ltd.); Trichloroacetic Acid (Tianjin Xinbute Chemical Co., Ltd.); Folinol (Beijing Boao Toda Science & Technology Co., Ltd.); Salicylic Acid (Tianjin Beichen Fangzheng Reagent Factory); Potassium Peroxydisulfate (Sangong Bioengineering Co., Ltd.); Ferrous Sulfate (Tianjin Henghsing Chemical Reagent Manufacturing Co., Ltd.); Ferric Chloride (Tianjin Henghsing Chemical Reagent Manufacturing Co., Ltd.); Methanol (Tianjin Komeo Chemical Reagent Co., Ltd.); p-Methoxyaniline, TBA, Rutin Standard, Gallic Acid Standard Ltd.); methanol (Tianjin Komeo Chemical Reagent Co., Ltd.); p-p-methoxyaniline,TBA, rutin standard, gallic acid standard, α-tocopherol standard, 2,4-dinitrophenylhydrazine, DPPH, ABTS reagent were purchased from McLean; petroleum ether, ether, 2,2-bipyridine, trichloromethane, isooctane, isopropanol, n-butanol, Sodium thiosulfate, anhydrous sodium sulfate, benzene solution were purchased from Tianjin Fuyu Fine Chemical Co. Sodium carbonate, glacial acetic acid, anhydrous ethanol, sodium hydroxide, potassium hydroxide were from Tianjin Tianli Chemical Reagent Co. All other reagents and drugs were analytically pure. The experimental water was deionised water.

### 2.2 Instruments and equipment

SpectraMax i3X Multifunctional Enzyme Labeler, Meigu Molecular Instrument Co., Ltd.; Digital Constant Temperature Water Bath, Xicheng Xinrui Instrument Factory, Jintan District; DV1MLVTJ0 Viscometer, Bohlefeld; esrE500 Electron Spin Resonance Spectrometer, Bruker, Germany; OXITEST Oil Oxidation Analyser, VELP Villeroy & Boch Instruments; DHG-9070 DHG-9070 Drying Oven, Changzhou Noki Instrument Co., Ltd.; Colour Difference Meter, Densho, Japan; PEN3 Electronic Nose, AIRSENSE, Germany; Thermo Trace 1300 Gas Chromatography-ISQ7000 Mass Spectrometry (GC–MS); Thermo Trace 1300 GC System, Thermo Fisher Scientific, USA.

### 2.3 Heating treatment of oil raw materials

Based on operational parameters from the production facility, four melting temperatures (50, 60, 70, and 80 °C) were selected for evaluation. Linear regression analysis of melting times for various oil volumes at these temperatures enabled extrapolation to industrial-scale processing (190 L). The predicted melting durations were 29, 24, 20, and 18 h at 50, 60, 70, and 80 °C, respectively.

### 2.4 Determination of oil quality indexes

#### 2.4.1 Determination of chromaticity

The chromaticity of the oil was determined by a colorimeter, and the values of L^⁎^, a^⁎^ and b^⁎^ were read, where L^⁎^ represents the colour range from black (0) to white (100), a^⁎^ represents the colour range from green (−) to red (+), and a^⁎^ represents the colour range from blue (−) to yellow (+), and the browning index was calculated (Chan et al., 2024).

#### 2.4.2 Determination of AV

The AV were measured according to official American Oil Chemists' Society (AOCS) methods Cd 5a-40(Qin et al., 2023).

#### 2.4.3 Determination of GCV

Prepare a 2,4-DNPH solution by dissolving 50 mg of 2,4-DNPH in 100 mL of 1-BuOH containing 3.5 mL of concentrated hydrochloric acid. Place 1 mL of oil sample into a 15 mL test tube and mix with 1 mL of 2,4-DNPH solution. Heat in a 40 °C water bath for 20 min. Dissolve potassium hydroxide (8 g) in 100 mL of 1-BuOH solution and add 8 mL of this solution. Centrifuge at 3000 rpm for 5 min and measure the absorbance of the supernatant at 420 nm(Liu et al., 2020).

### 2.5 Determination of bioactive components of oil

#### 2.5.1 Determination of flavonoid content

Take 10 mL of oil, according to the material-liquid ratio of 1:10 g/mL to add 80 % ethanol solution. 80 °C water bath for 100 min, centrifuged at 4000 r/min centrifuge for 10 min, and take the supernatant at low temperature and protected from light to save, to be used. Using rutin as the standard, the standard solution of rutin was prepared at a mass concentration of 0.2 mg/mL, 0, 0.5, 1.0, 1.5, 2.0 and 2.5 mL of rutin standard solution was accurately sucked up into a 10 mL cuvette with a pipette. Then anhydrous ethanol was added into the 10 mL cuvette, and then 5 g/100 mL NaNO_2_ solution was added into the 10 mL cuvette, and then 5 g/100 mL Al(NO_3_)_3_ solution was added into the 10 mL cuvette and then 10 g/100 mL Al(NO_3_)_3_ solution was added into the cuvette. Then add 10 g/100 mL of Al(NO_3_)_3_ solution 0.5 mL, shake well, let stand for 6 min; finally add 5 g/100 mL of NaOH solution 4 mL, with anhydrous ethanol solution fixed to the scale, shaking well, let stand for 20 min; the absorbance value at 510 nm wavelength was measured. Pipette 3 mL of the filtrate according to the above method, and then calculate the mass concentration of flavonoids according to the standard curve(Tohidi et al., 2017).

#### 2.5.2 Determination of total phenol content

According to(Ahmed & Tavaszi-Sarosi, 2019), take 3 mL of oil add 45 mL of 70 % ethanol, extract at 60 °C, centrifuged at 4000 r/min for 15 min, and take gallic acid as the standard, prepare the mass concentration of 10 mg/mL standard solution, diluted according to a certain gradient, take 1 mL add 10 % by volume forintol colour developer 5 mL, mix well, add 7.5 g/100 mL of sodium carbonate solution 4 mL in each of the 3–8 min, mix well and leave it for 1 h at room temperature, protected from light, and then measure absorbance value at 765 nm. Pipette 1 mL of the test solution into a 10 mL centrifuge tube, determine the absorbance at 765 nm according to the above method, and then calculate the mass concentration of total phenols in oil according to the standard curve.

#### 2.5.3 Determination of alpha-tocopherol content

Take 200 mg of oil sample in a 10 mL volumetric flask, add 5 mL of trichloromethane, mix well, then add 3.5 mL of 2,2′-bipyridine (0.7 mg/mL) and 0.5 mL of FeCl_3_ (2 mg/mL), and the volume fraction of 95 % ethanol was fixed to 10 mL, and then left to stand for 1 min, and then the absorbance was measured at 520 nm using a blank of reagent without oil sample. The tocopherol content in the oil was calculated from the standard curve(Ciuffarin et al., 2023).

#### 2.5.4 Determination of β-carotene content

1 g of oil was mixed with 5 mL of an extraction solution containing hexane:acetone:ethanol (70,15,15) at 25 °C with shaking(Mostafa Al-Turky et al., 2024). Then 0.1 mL of methanol solution of KOH (40 % *w*/*v*) was added to the mixture and saponified for 2 h at 25 °C. Then 5 mL of hexane was added to the mixture. The mixture was centrifuged at 5000 rpm for 30 min and the carotenoid extract was recovered. The residual layer was extracted again using 3 mL of hexane. The total carotenoid content of the extract was determined using a spectrophotometer. Calculations were performed using a standard curve of β-carotene in hexane solution (5–60 mg/L) at 450 nm.

### 2.6 Determination of antioxidant capacity of oil

#### 2.6.1 DPPH free radical scavenging rate measurement

A certain mass of oil sample was dissolved in methanol and prepared into different mass concentrations of sample solution. 0.1 mM of DPPH-methanol solution was prepared in methanol and mixed with 1 mL of sample solution(Adeosun et al., 2013). The reaction was carried out at room temperature and protected from light for 30 min. The absorbance of the sample solution (A₁) and the methanol blank (A₀) were measured at 518 nm. The DPPH radical scavenging rate was calculated using Eq.1.

(1) $$\text{DPPH}\left(\%\right)=\left(\frac{A_{0}-A_{1}}{A_{0}}\right)\times 100\%$$

#### 2.6.2 Measurement of hydroxyl radical scavenging rate

According to a previously reported method(Lu et al., 2018). 2 mL of the oil sample was mixed with 2 mL of phosphate buffer solution (0.2 M, pH 7.4), 1 mL of phenanthroline (0.75 × 10^−3^ M), 1 mL of FeSO_4_ (0.75 × 10^−3^ M), and 1 mL of 0.3 % H_2_O_2_, and the mixture was heated at 37 °C for 30 min. The absorbance (A_1_) was then measured at 510 nm. A blank (A_0_) was prepared by replacing the oil with an equal volume of distilled water. The hydroxyl radical scavenging rate was calculated by replacing the FeSO_4_ solution (A_i_) with deionised water as shown in Eq. 2.

(2) $$\begin{array}{c} \text{hydroxyl}\left(\%\right)=\left(\frac{1-A_{1}+A_{i}}{A_{0}}\right)\times 100\% \end{array}$$

#### 2.6.3 ABTS free radical scavenging rate measurement

According to a previously reported method (Mostafa Al-Turky et al., 2024). The oil was dissolved in anhydrous ethanol, 0.5 mL of which was added to 1 mL of ABTS working solution (1 mL of 7 mmol/L ABTS solution mixed with 1 mL of 2.45 mmol/L potassium persulfate solution), and the reaction was carried out at 25 °C and protected from light for 16 h. The sample was then diluted in anhydrous ethanol to an absorbance at 734 nm, and the absorbance A_1_ was measured at 734 nm after 30 min reaction at room temperature. The blank group was anhydrous ethanol instead of the sample, A_0_. The calculation of the radical scavenging rate of ABTS is shown in Eq. 3.

(3) $$\begin{array}{c} \text{ABTS}\left(\%\right)=\left(\frac{A_{0}-A_{1}}{A_{0}}\right)\times 100\% \end{array}$$

### 2.7 Determination of oxidative properties of oil

#### 2.7.1 Determination of total free radicals

According to a previously reported method(Sun et al., 2024), with some modifications. 20 μL of 5,5-dimethyl-1-pyrroline N-oxide(DMPO) was added to an appropriate amount of oil sample and placed in the resonance chamber of an electron spin resonator. The instrument parameters were set as follows: power of 3.99 mW, band 9.2 to 9.8 GHz, scan width of 100 G, modulation frequency of 100 kHz; transition time of 1.28 ms; time constant of 20.48 ms. The resonant cavity was heated at 60 °C, and the detection times were 10 min, 20 min, 30 min and 40 min, respectively.

#### 2.7.2 Determination of oxidation induction period

The oxidative stability of the samples was evaluated using an OXITEST Fat Oxidation Analyser. The induction period was determined under the following conditions: reaction cell temperature maintained at 100 °C, pressure at 6 bar, and sample mass of 10 g(Kreps et al., 2021).

#### 2.7.3 Determination of CD and conjugated trienes

According to a previously reported method(Kreps et al., 2021). 0.01 to 0.03 g of the oil sample was weighed into a 25 mL volumetric flask and diluted to scale with isooctane (spectrophotometric grade). The absorbance at the indicated wavelengths was then measured at 233 nm and 268 nm.

(4) $$\begin{array}{c} C_{\mathrm{CD}}=\frac{A}{\mathrm{\varepsilon l}} \end{array}$$

(5) $$\begin{array}{c} \mathrm{CD}\;\text{value}=\frac{C_{\mathrm{CD}}}{W}\times 2.5\times 10^{4}\# \end{array}$$

where l is the path length of the cuvette in centimetres; A is the absorbance of the sample at 233 nm and 268 nm for CD and conjugated trienes(CT); ε is the molar absorptivity of hydrogen peroxide from linoleic acid; 2.5 × 10^4^ is a coefficient and W is the weight of the sample in grams.

#### 2.7.4 Determination of peroxide value

Weigh the oil about 2.5 g, add 35 mL trichloromethane-ice acetic acid (trichloromethane: ice acetic acid mixture = 2:3) mixture, and then add 1 mL of potassium iodide saturated solution, shaking for 30 s, in the dark for 4 min. Add 75 mL of water, and shake well. Add 1 mL of 0.5 % starch indicator, titrate with sodium thiosulfate standard solution at a concentration of about 0.002 mol/L until the black colour disappears, and record the volume of sodium thiosulfate standard solution used(Sun et al., 2024). The peroxide value results(POV) were calculated using Eq. 6.

(6) $$\begin{array}{c} P=\frac{1000\left(V-V_{0}\right)C}{2m} \end{array}$$

Where V is the volume of sodium thiosulphate standard solution consumed in the titration;V_0_ is the volume of sodium thiosulphate standard solution used for blank; C is the concentration of sodium thiosulphate standard solution; m is the mass of the sample.

#### 2.7.5 Determination of anisidine value

The anisidine value(p-AV) was determined based on the ISO 6885 method(Rajagukguk et al., 2024).

#### 2.7.6 Determination of 2-thiobarbituric acid value

According to a previously reported method (Esfahani et al., 2024). 200 mg of the oil sample was transferred to a 25 mL centrifuge tube and 1-butanol was added to the scale. 5 mL of the above solution and 10 mL of 0.2 % 2-thiobarbituric acid(TBA) reagent were added. 5 mL of the above solution and 10 mL of 0.2 % TBA reagent were added and the tube was placed in a water bath at 95 °C for 2 h. The tube was placed in cold water until it reached room temperature. The TBA content was calculated as shown in Eq. 7.

(7) $$\begin{array}{c} \mathrm{TBA}=50\times \frac{A-B}{m} \end{array}$$

Where A is the absorption of the sample at 532 nm, B is the control absorption at 532 nm m is the weight of the sample (g).

#### 2.7.7 Determination of fatty acid composition

Chromatographic conditions(Hoving et al., 2018): Chromatographic column Thermo TG-FAME capillary column (50 m × 0.25 mm 1D × 0.20 μm); shunt injection, injection volume of 1 mL, shunt ratio of 8:1. 250 °C inlet temperature; ion source temperature 300 °C; the temperature of the transmission line 280 °C. The starting temperature was 80 °C, held for 1 min; 20 °C/min to 160 °C, held for 1.5 min; 3 °C/min to 196 °C, held for 85 min; and finally 20 °C/min to 250 °C, held for 3 min. The carrier gas was helium, and the flow rate of the carrier gas was 0.63 mL/min.

Mass Spectrometry Condition: electron bombardment ionisation (EI) source, SIM scanning mode, electron energy 70 eV.

### 2.8 Determination of oil flavor substances

#### 2.8.1 *E*-nose

A 5 mL oil sample was taken from a 20 mL headspace vial, equilibrated for 10 min at room temperature using a PEN3 electronic nose, sample measurement interval 1 s; sample testing time 70 s; measurement and counting time 1 s; and internal and inlet flow rates 300 mL/min. The data were taken throughout 57–59 s, and each sample was measured(Liu et al., 2024). The sensitive volatile compounds for each sensor are: W1C is sensitive to aromatic compounds; W5S is sensitive to nitrogen oxides; W3C is sensitive to aromatic compounds and ammonia; W6S selects primarily hydrides; W5C is sensitive to olefins and aromatic compounds; W1S is sensitive to alkanes; W1W is sensitive to sulfides; W2S is sensitive to alcohols, aldehydes, and ketones; W2W is sensitive to organosulfides and aromatic compounds, and W3S is sensitive to organic sulfur compounds. W2W is sensitive to organosulfides and aromatic compounds, and W3S is sensitive to long-chain alkanes and aliphatics.

#### 2.8.2 Determination of volatile flavor substances

1.5 mL of the oil sample was taken in a headspace vial and 500 mg of 2-methyl-3-heptanone was added as an internal standard(Zhang et al., 2023). Chromatographic separation conditions: Column: DB-wax; injection temperature: 230 °C; split ratio: 10:1, carrier gas: helium (99.999 %); flow rate: 1.0 mL/min; column temperature: initial temperature 50 °C for 4 min, increase to 230 °C at 5 °C/min and keep for 5 min; Ion source temperature: 230 °C; ionisation mode: 230 °C; Ion source temperature: 230 °C; Scanning mode: full scan; Mass range: 33–550; NIST 2017 Spectral Library. SPME conditions:Equipment: CTC Trinity autosampler, extraction head: 65 μm PDMS/DVB 1 cm, temperature: 50 °C, time: shaking 15 min, extraction 30 min, shaking speed: 250 rpm, resolution time: 5 min, GC cycle time: 50 min.

### 2.9 Statistical analysis

The data were presented as mean ± standard deviation (SD) for each technical repeat. One-way analysis of variance (ANOVA) was analyzed using SPSS 26.0 software (IBM SPSS Inc., Chicago, USA). GraphPad Prism 9 (GraphPad Software, Inc., San Diego, USA) and Origin 2021 (Origin Lab Inc., USA) were used to generate the graphs.

## 3 Results and discussion

### 3.1 Oil quality

#### 3.1.1 Chromaticity

**Fig. 1:**
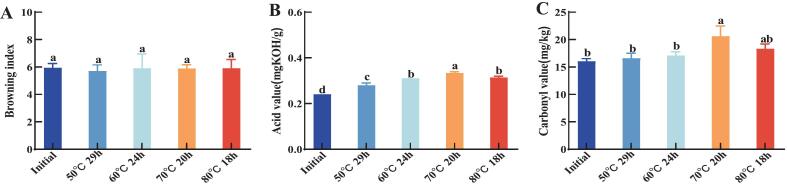
Changes in quality of oil after melting at different temperatures and times. (A) is the colour of the raw oil and fat; (B) is the acid value of the raw oil and fat; (C) is the carbonyl value of the raw oil and fat. Respectively. Different letters for the same index mean significant difference (*p* < 0.05).

As shown in Fig. 1A, the browning indices of the oil after melting by four different melting processes ranged from 5.94 to 5.70, and there was no significant difference in the degree of browning of the oil after melting by different processes compared with the initial oil. Lipid oxidation reactions in oil produce specific products, which may darken the colour of oil (Zeng et al., 2024). However, it is difficult to reflect the variability in the colouration of the oil raw materials due to the overall low and small differences in the oil melting temperatures.

#### 3.1.2 Av

The AV of oil represents the amount of free fatty acids it contains, reflecting the degree of oxidation changes. The results of this experiment are shown in Fig. 1B. The initial value of the AV of the oil raw materials was 0.24 (mg KOH/g), and the AV of the oil raw materials increased to different degrees under the four melting processes. The lowest AV value was obtained after 29 h of treatment at 50 °C, which was 0.28 (mg KOH/g), an increase of only 0.04 (mg KOH/g) compared to the initial sample. The highest AV value was 0.33 (mg KOH/g), which was measured after 20 h of treatment at 70 °C. The increase in the AV was due to the hydrolysis or lipolysis leading to the decomposition of triacylglycerol, in which free fatty acids were formed(Sun et al., 2021a).

#### 3.1.3 GCV

The GCV reflects the total amount of aldehydes, ketones and other substances produced during the oxidation process of the raw material. The results of GCV of oil raw material after different melting processes are shown in Fig. 1C. The initial oil exhibited a GCV of 16.057 mg/kg. Following thermal treatment at 50 °C for 29 h and 60 °C for 24 h, the GCV increased marginally to 16.59 and 17.10 mg/kg, respectively, with no statistically significant difference (*p* > 0.05) compared to the untreated sample. The GCV was the highest in the 70 °C for 20 h process at 20.62 mg/kg followed by 18.30 mg/kg at 80 °C for 18 h. Which represented a serious deterioration of the quality of oil under the two higher temperature processes, which is similar to the results of Maria Kasprzak et al. (Kasprzak et al., 2020).

### 3.2 Bioactive components of oil

**Fig. 2:**
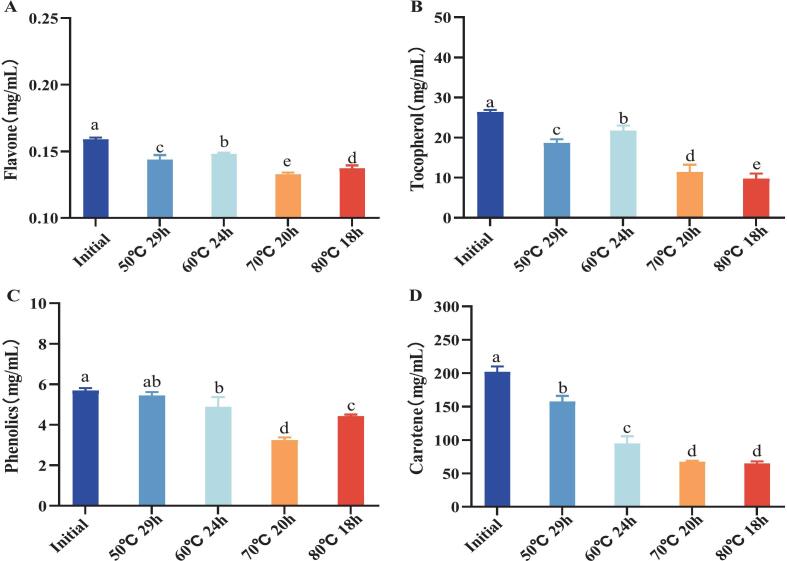
Changes in antioxidant capacity of oil after melting at different temperatures and times. (A) DPPH radical scavenging rate; (B) hydroxyl radical scavenging rate; (C) ABTS radical scavenging rate. Respectively. Different letters for the same index mean significant difference (p < 0.05).

The vegetable oil raw material of infant formula milk powder is a mixed vegetable oil. In addition to the main ingredient OPO, there are also two vegetable oils, soybean oil and corn oil, both containing bioactive ingredients such as flavonoids, carotene and tocopherol. In addition, mixed tocopherol is added as an antioxidant to inhibit oxidation of vegetable oil. Bioactive ingredients are prone to degradation at high temperatures, and their antioxidant ability is also affected by temperature and concentration. Therefore, exploring the changes in bioactive ingredients in vegetable oil raw materials after heat treatment plays an important role in maintaining the nutritional components of milk powder. The changes in the bioactive components of vegetable oil after the four heat treatments are shown in Fig. 2. The contents of flavone, phenolics, tocopherol and carotene in untreated vegetable oils were 0.15 mg/mL, 5.68 mg/mL, 26.34 mg/mL and 201.77 mg/mL, respectively. The contents of the four bioactive components decreased by the maximum after the heat treatment: carotene 68 % > tocopherol 63 % > tocopherol 42 % > flavonoids 18 %, which also showed the difference in the thermal sensitivity of the four bioactive components. After heating treatment, the content of flavonoids, total phenols, α-tocopherols and carotene decreased by up to 0.027 mg/mL, 2.44 mg/mL, 16.61 mg/mL and 137.22 mg/mL, and both occurred under the higher temperature heat treatments of 70 °C for 20 h, 80 °C for 18 h. This indicates that the bioactive ingredients in vegetable oil are more sensitive to temperature(Mehany et al., 2025).

### 3.3 Antioxidant capacity of oil

**Fig. 3:**
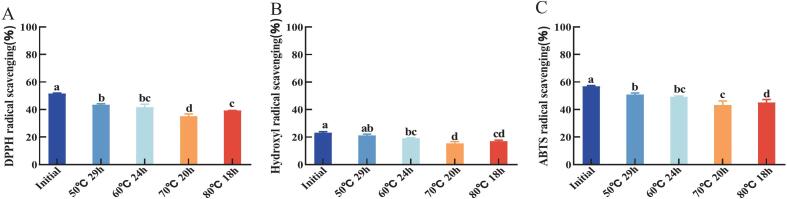
Changes in oxidative stability of oil after melting at different temperatures and times. Where (a) oxidation induction period; (b) conjugated diene content; (c) conjugated triene content; (d) peroxide value; (e) anisidine value; (f) malondialdehyde content.respectively. Different letters for the same index mean significant difference (p < 0.05).

Antioxidant capacity is essential for assessing the quality of oil. This experiment determined the antioxidant capacity of vegetable oil raw materials in infant formula using three methods. The results are shown in Fig. 3. The scavenging rates of DPPH radicals, hydroxyl radicals and ABTS radicals by the initial oil were 51.61 %, 23.16 % and 56.83 %, respectively. After treatment with four melting processes, it can be seen a reduction in all components with antioxidant capacity in the oil treated with melting processes(El Idrissi et al., 2023). Similarly, the three radical scavenging rates of the oil after melting at 50 °C for 29 h and 60 °C for 24 h processes decreased less compared with those after melting at 70 °C for 20 h and 80 °C for 18 h processes, with the lowest decreases of the three radical scavenging rates at 50 °C for 29 h processes, which decreased by 8.23 %, 2.04 % and 6.05 %, respectively. The 70 °C for 20 h treatment resulted in greater reduction in radical scavenging capacity compared to the 80 °C for 18 h process. Specifically, scavenging rates decreased by 16.6 % for DPPH radicals, 7.84 % for hydroxyl radicals, and 13.61 % for ABTS radicals. This enhanced degradation may be attributed to the prolonged thermal exposure duration.

### 3.4 Oxidative properties of oil

#### 3.4.1 Total free radicals

**Fig. 4:**
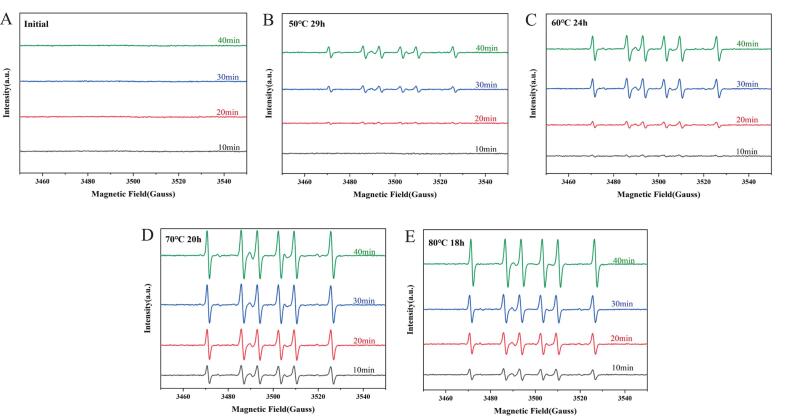
Changes of free radical peak intensities of oil at different melting processes. (A) is the free radical peak intensity of initial oil; (B) is the free radical peak intensity of oil at 50 °C for 29 h; (C) is the free radical peak intensity of oil at 60 °C for 24 h; (D) is the free radical peak intensity of oil at 70 °C for 20 h; (E) is the free radical peak intensity of oil at 80 °C for 18 h.

A large number of free radicals will be generated during the oxidation process of oil raw materials. The degree of oxidation of oil was assessed by determining the strength of free peaks of the oil. The results are shown in Fig. 4. DMPO was used as a capture agent for free radical species identification and quantification, the free radicals generated from the oil after different melting processes are alkoxy radicals(Yang et al., 2022), which combined with hydrogen atoms to be converted to hydroperoxides(Yin et al., 2011). Alkyl radicals are radicals produced by the dehydrogenation reaction of the α-position of unsaturated fatty acids and The C—H bond energy of the α-position of oleic acid and linoleic acid is low, and the oil contains high levels of oleic acid and linoleic acid. These reasons may account for the higher number of alkyl radicals in the heated oil (Chen et al., 2021). The results are shown in Fig. 4. No significant radical signals were detected for the initial oil, and alkyl radicals were detected in the samples treated by the four oleochemical processes. From the plots, it can be concluded that the oil at 50 °C for 29 h process has the lowest peak intensity and the least amount of free radicals. The peak intensities of the free radical profiles at the lower two temperatures were lower than those at the higher temperatures, representing their lower degree of oxidation.

#### 3.4.2 Oxidation induction period

**Fig. 5:**
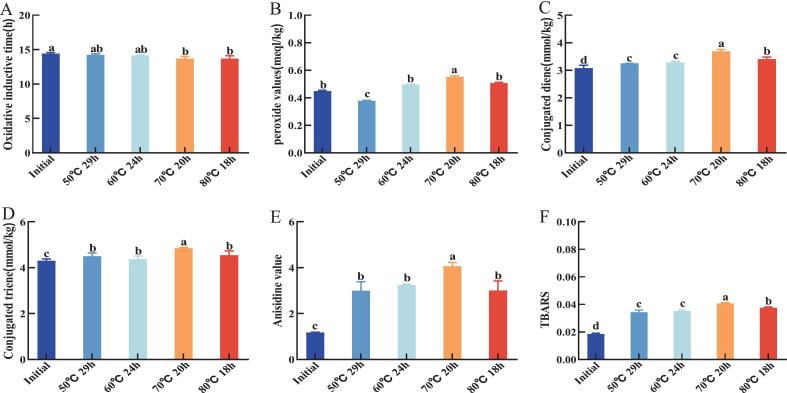
Changes in oxidative stability of oil after melting at different temperatures and times. Where (a) oxidation induction period; (b) conjugated diene content; (c) conjugated triene content; (d) peroxide value; (e) anisidine value; (f) malondialdehyde content. Respectively. Different letters for the same index mean significant difference (*p* < 0.05).

As shown in Fig. 5A, the oxidation induction period of the initial oil is 14.4 h. The longer the oxidation induction period, the better the oxidative stability of the oil. After different melting processes, the oxidation induction periods at 50 °C, 29 h and 60 °C for 24 h were not significantly different from the initial samples. The decrease in the oxidation induction periods at 70 °C for 20 h and 80 °C for 18 h was due to the further rapid generation of primary and secondary products of lipid oxidation (Rajagukguk et al., 2024). This indicates that high temperatures have a greater impact on the oxidation induction period; the higher the temperature, the more significant the decline in oxidation stability.

#### 3.4.3 POV

Peroxides are the primary products of fat oxidation. Peroxides are unstable and will further decompose to produce aldehydes, ketones. The results of this experiment are shown in Fig. 5B; the initial oil has the lowest POV of 0.448 meq/kg, which represents its freshness and long shelf life(Yadav et al., 2018). The highest value of 0.552 meq/kg was observed at 70 °C for 20 h. Due to the catalytic action of unsaturated fatty acids in oil at high temperatures and under other conditions, unstable free radicals are produced. These free radicals then react further with fatty acid molecules to form peroxides, leading to an increase in POV values.

#### 3.4.4 CD and CT

The results of this experiment are shown in Fig. 5C and D. The initial CD and CT values were 3.07 mmol/kg and 4.29 mmol/kg, respectively, and the CD and CT of the oil raw materials were elevated to some extent after treatment with the four different melting processes(Rakariyatham et al., 2024). The CD value of the oil raw materials at 50 °C for 29 h was the least elevated at 3.24 mmol/kg, and the CT value of the oil raw materials at 60 °C for 24 h was the least elevated at 4.37 mmol/kg. The CD and CT values were the highest under the conditions of 70 °C for 20 h, with the values of 3.68 mmol/kg and 4.85 mmol/kg, representing their higher oxidation rates and levels of oxidation products.

#### 3.4.5 P-AV

The p-AV is an indicator of the content of secondary products during the oxidation of oil(Zhang et al., 2010). The results of this experiment are shown in Fig. 5E. The p-AV of the initial oil sample was 1.17, and the p-AV of the oil increased to different degrees after four different melting processes(Zhao et al., 2021). The lowest p-AV increase (2.99) was observed following treatment at 50 °C for 29 h. There was no significant difference between the p-AV at 50 °C for 29 h, 60 °C for 24 h, and 80 °C for 18 h. The reason was that less peroxide was generated at these three temperatures, therefore fewer aldehydes were decomposed. The highest p-AV of 4.056 was found at 70 °C for 20 h, both of which represent a higher degree of oxidation of the oil at 70 °C for 20 h process.

#### 3.4.6 TBA

The initial oxidation products of the oil further decomposed to form malondialdehyde. The results of this experiment are shown in Fig. 5F. The initial TBA value of the oil was 0.018, and the TBA values increased to different degrees after four different temperatures and times of treatment. The maximum TBA value was 0.04 at 70 °C for 20 h process, followed by 0.038 at 80 °C for 18 h process, and the lowest TBA value was 0.034 at 50 °C for 29 h process. There was no significant difference between the TBA values at 50 °C for 29 h and 60 °C for 24 h process.

#### 3.4.7 Fatty acid composition

As shown in Table 1, 15 saturated fatty acids **(SFA)**, 21 unsaturated fatty acids **(UFA)**, and 11 trans fatty acids **(TFA)** were detected in the oil. In the initial oil SFA, UFA and TFA accounted for 34.86 %, 64.69 % and 0.43 %, respectively. Among them, palmitic acid, oleic acid, linoleic acid and linolenic acid had the highest content of several fatty acids. After treatment with the four different melting processes, the content of saturated and UFA decreased to varying degrees. The content of SFA decreased with the increase of heating temperature, which shows that high temperature is more likely to cause the decomposition of SFA. The unsaturated fatty acid content decreased in the order of 50 °C for 29 h (5603 μg/mL); 60 °C for 24 h (4453 μg/mL); 70 °C for 20 h (7880 μg/mL), and 80 °C for 18 h (7767 μg/mL) after treatment with the four oleochemical processes(Khaled et al., 2024). Unsaturated fats are highest in oleic acid, followed by linoleic and linolenic acids. These three fatty acids decreased most significantly with increasing temperature(Sun et al., 2021b).

**Table 1:** Changes in fatty acid content of oil after treatment with different carburetion processes.

Fatty Acid	Initial (μg/mL)	60 °C 24 h (μg/mL)	70 °C 20 h (μg/mL)	50 °C 29 h (μg/mL)	80 °C 18 h (μg/mL)
C6:0	0.52 ± 0.02^a^	0.50 ± 0.01^a^	0.57 ± 0.03^a^	0.75 ± 0.02^a^	0.71 ± 0.01^a^
C8:0	13.21 ± 0.15^b^	11.63 ± 0.08^a^	13.72 ± 0.05^b^	12.71 ± 0.04^a^	13.89 ± 0.31^b^
C10:0	6.96 ± 0.12^a^	6.12 ± 0.09^a^	7.26 ± 0.01^b^	6.82 ± 0.05^a^	7.3 ± 0.19^b^
C12:0	60.08 ± 0.11^b^	52.75 ± 0.34^a^	63.8 ± 0.49^c^	58.22 ± 0.30^b^	61.63 ± 0.46^bc^
C13:0	0.86 ± 0.01^a^	0.86 ± 0.01^a^	0.9 ± 0.01^a^	0.88 ± 0.01^a^	0.86 ± 0.02^a^
C14:0	273.97 ± 0.92^d^	218.65 ± 1.07^a^	239.9 ± 3.72^b^	225.81 ± 2.90^a^	257.52 ± 2.82^c^
C15:0	15.83 ± 0.05^a^	14.62 ± 0.08^a^	18.26 ± 0.05^b^	14.95 ± 0.01^a^	16.02 ± 0.05^a^
C16:0	16,156.65 ± 125.92^e^	15,905.13 ± 210.14^d^	15,452.15 ± 51.69^c^	14,762.83 ± 57.68^b^	14,582.28 ± 3.51^a^
C17:0	36.78 ± 0.87^a^	33.35 ± 0.13^a^	42.38 ± 0.12^b^	34.21 ± 0.01^a^	36.41 ± 0.03^a^
C18:0	2271.1 ± 21.43^e^	2004.5 ± 10.7^d^	1957.3 ± 41.2^c^	1870.4 ± 26.8^b^	1810.8 ± 9.71^a^
C20:0	126.6 ± 0.19^c^	112.23 ± 4.85^b^	106.72 ± 0.72^a^	101.05 ± 1.73^a^	102.8 ± 0.23^a^
C21:0	7.44 ± 0.34^c^	6.47 ± 0.31^ab^	6.89 ± 0.01^b^	6.2 ± 0.08^a^	5.83 ± 0.1^a^
C22:0	102.1 ± 0.18^c^	78.52 ± 1.48^a^	80.16 ± 2.22^ab^	77.57 ± 1.55^a^	83.36 ± 0.2^b^
C23:0	23.6 ± 0.54^b^	14.09 ± 0.28^a^	15.21 ± 0.48^a^	16.6 ± 0.33^a^	14.45 ± 0.39^a^
C24:0	42.73 ± 0.25^c^	34.31 ± 0.29^a^	36.2 ± 0.22^a^	36.09 ± 0.33^a^	39.36 ± 1.04^b^
SFA	19,138.44	18,493.74	18,041.47	17,225.09	17,033.29
C14:1	3.02 ± 0.15^c^	2.41 ± 0.15^b^	2.5 ± 0.03^b^	1.46 ± 0.05^a^	2.37 ± 0.11^b^
C15:1	2.87 ± 0.08^b^	2.64 ± 0.06^b^	2.79 ± 0.13^b^	1.68 ± 0.02^a^	2.72 ± 0.08^b^
C16:1	46.71 ± 0.21^b^	38.4 ± 0.18^a^	41.59 ± 0.25^a^	38.67 ± 0.43^a^	40.12 ± 0.07^a^
C17:1	13.5 ± 0.51^b^	11.53 ± 0.21^a^	12.63 ± 0.39^a^	11.17 ± 0.19^a^	10.81 ± 0.04^a^
C20:1	774.69 ± 9.88^d^	633.33 ± 4.29^b^	701.05 ± 7.43^c^	601.6 ± 17.63^a^	624.1 ± 12.15^b^
C24:1	9.8 ± 0.27^b^	10.57 ± 0.15^c^	10.73 ± 0.33^c^	6.22 ± 0.4^a^	8.05 ± 0.07^b^
C18:1 N9C	17,457.17 ± 800.8^e^	15,320.02 ± 173.38^c^	16,368.19 ± 1305.95^d^	13,921.36 ± 1760.2^a^	14,210.64 ± 1344.61^b^
C18:1 N7	298.26 ± 0.95^d^	235.01 ± 0.78^b^	257.49 ± 2.97^c^	214.47 ± 0.01^a^	234.74 ± 3.07^b^
C18:2 N6	15,590.78 ± 93.10^e^	13,438.03 ± 279.41^d^	13,348.79 ± 158.30^c^	12,706.87 ± 85.59^a^	12,467.30 ± 27.22^a^
C18:3 N6	11.10 ± 0.80^b^	9.51 ± 0.28^a^	10.50 ± 0.49^a^	9.36 ± 0.31^a^	9.50 ± 0.02^a^
C18:3 N3	2077.73 ± 29.30^e^	1685.10 ± 1.15^c^	1852.06 ± 4.90^d^	1559.30 ± 1.00^a^	1602.60 ± 36.20^b^
C20:3 N6	8.14 ± 0.37^a^	8.06 ± 0.25^a^	8.05 ± 0.26^a^	7.60 ± 0.38^a^	7.57 ± 0.01^a^
C22:1 N9	10.17 ± 0.35^b^	9.37 ± 0.12^a^	8.92 ± 0.16^a^	8.78 ± 0.22^a^	8.92 ± 0.39^a^
C20:3 N3	8.87 ± 0.16^a^	8.12 ± 0.19^a^	8.79 ± 0.43^a^	8.26 ± 0.08^a^	8.12 ± 0.16^a^
C20:4 N6	10.95 ± 0.40^a^	10.30 ± 0.34^a^	10.56 ± 0.75^a^	10.77 ± 0.13^a^	10.46 ± 0.25^a^
C22:2	9.66 ± 0.04^a^	9.21 ± 0.24^a^	9.07 ± 0.31^a^	8.68 ± 0.24^a^	8.59 ± 0.53^a^
C20:5 N3	5.60 ± 0.05^a^	5.28 ± 0.24^a^	5.57 ± 0.27^a^	5.15 ± 0.06^a^	4.98 ± 0.23^a^
C22:4	6.89 ± 0.2^a^	6.46 ± 0.18^a^	6.46 ± 0.02^a^	6.38 ± 0.19^a^	6.61 ± 0.17^a^
C22:5 N6	6.37 ± 0.18^a^	6.12 ± 0.15^a^	6.24 ± 0.40^a^	6.09 ± 0.25^a^	6.10 ± 0.26^a^
C22:5 N3	6.13 ± 0.08^a^	5.37 ± 0.1^a^	5.83 ± 0.27^a^	5.63 ± 0.21^a^	5.84 ± 0.10^a^
C22:6 N3	6.69 ± 0.4^a^	6.26 ± 0.08^a^	6.13 ± 0.27^a^	6.45 ± 0.24^a^	6.64 ± 0.03^a^
UFA	36,365.07	30,762.23	31,912.65	28,485.15	28,598.74
C14:1 T	1.09 ± 0.11^a^	1.44 ± 0.07^a^	1.57 ± 0.04^a^	1.93 ± 0.10^b^	2.00 ± 0.07^b^
C15:1 T	1.71 ± 0.07^a^	2.50 ± 0.18^b^	2.58 ± 0.05^b^	3.00 ± 0.2^b^	2.90 ± 0.01^b^
C16:1 T	2.77 ± 0.09^a^	3.48 ± 0.19^b^	2.63 ± 0.29^a^	4.47 ± 0.06^d^	4.08 ± 0.01^c^
C17:1 T	3.87 ± 0.16^a^	4.8 ± 0.02^b^	4.45 ± 0.06^b^	4.99 ± 0.18^b^	5.12 ± 0.05^c^
C18:1N12T	52.39 ± 0.49^a^	47.37 ± 0.74^b^	49.86 ± 2.08^a^	47.03 ± 0.38^b^	49.05 ± 1.58^a^
C18:1 N9T	55.90 ± 0.38^a^	54.75 ± 0.32^a^	51.55 ± 0.32^a^	55.18 ± 0.53^a^	44.24 ± 0.81^b^
C18:1 N7T	36.48 ± 1.57^a^	39.03 ± 0.73^b^	40.67 ± 1.24^b^	38.45 ± 2.41^b^	36.81 ± 0.04^a^
C18:2 N6T	11.9 ± 0.05^a^	11.69 ± 0.22^a^	11.86 ± 0.17^a^	15.12 ± 0.42^b^	15.31 ± 0.15^b^
C19:1 N9T	74.41 ± 1.11^b^	77.41 ± 1.33^c^	75.43 ± 2.09^b^	68.94 ± 0.07^a^	74.04 ± 1.66^b^
C20:1 T	9.06 ± 0.59^a^	10.62 ± 0.03^b^	10.46 ± 0.19^b^	13.54 ± 0.33^c^	11.29 ± 0.02^b^
C22:1 N9T	6.27 ± 0.48^a^	8.19 ± 0.03^b^	7.24 ± 0.09^a^	9.54 ± 0.14^c^	9.43 ± 0.10^c^
TFA	255.85	261.27	258.31	262.21	254.27

Respectively. Different letters for the same index mean significant difference (*p* < 0.05).

### 3.5 Oil flavor substance

#### 3.5.1 *E*- nose

**Fig. 6:**
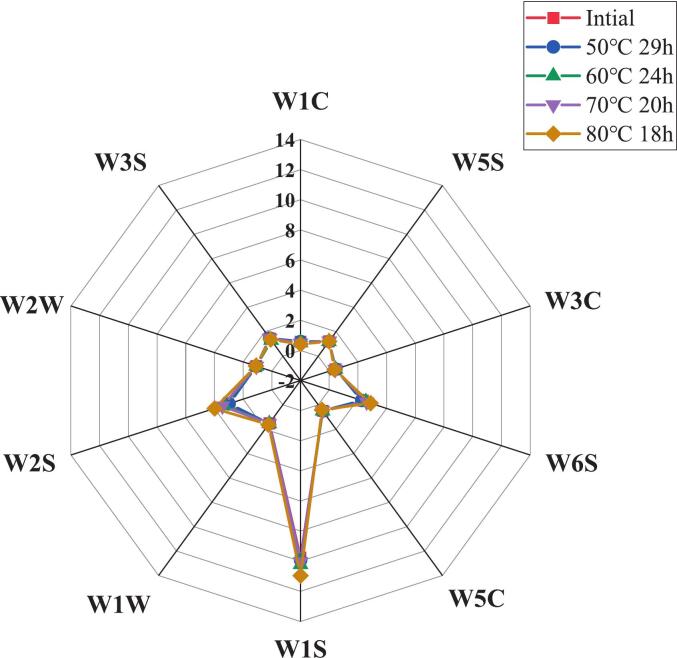
*E*-nose profiles of oil after treatment with different carburetion processes.

The use of electronic nose can effectively evaluate the overall flavor substances of infant formula oil raw materials under different melting processes. As can be seen in Fig. 6, the differences substance in flavor between the oil treated at different temperatures and times and the initial samples are mainly reflected in the W1S(alkanes), W6S (hydrides) and W2S (alcohols, some aromatic compounds). From the Fig, it can be seen that the three types of volatile flavor substances related to lipid oxidation in the oil under 80 °C for 18 h treatment were most obviously elevated, followed by the oil under 70 °C for 20 h, which represented a higher degree of oxidation of the oil under the two temperature treatments The difference between the oil under 50 °C for 29 h and 60 °C for 24 h and the initial sample had a higher degree of overlap in the e-nose profile.

#### 3.5.2 Volatile flavor substances

**Fig. 7:**
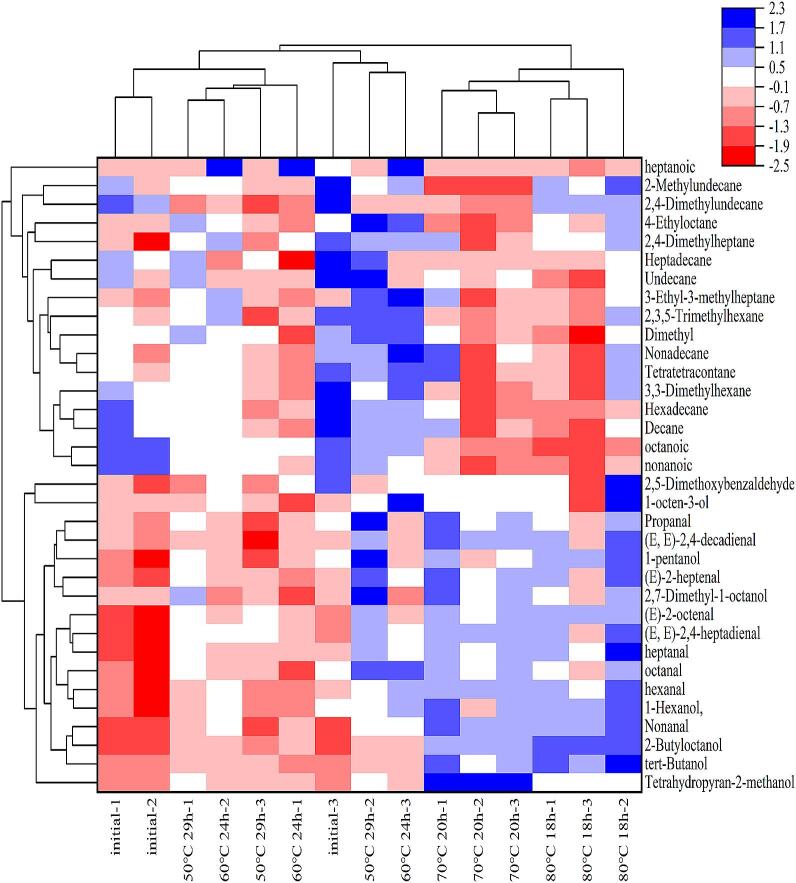
Thermograms of flavor substances of oil after treatment with different carburetion processes.

In order to solve the problem of the electronic nose in accurately characterising and quantifying, the present experiments further analyzed the volatile flavor substances of oil using GC–MS(Zhao et al., 2025). The results are shown in Table 2 and Fig. 7 alcohols, 10 aldehydes, 3 acids and 13 alkanes were detected in the oil raw materials.

**Table 2:** Changes of volatile flavor substances in oil after different melting processes.

Compounds	Relative amount (%)				
	initial	50 °C 29 h	60 °C 24 h	70 °C 20 h	80 °C 18 h
Alcohols (7)					
Tetrahydropyran-2-methanol	0.66 ± 0.03^a^	1.60 ± 0.06^b^	1.22 ± 0.10^b^	3.20 ± 0.01^c^	1.64 ± 0.02^b^
tert-Butanol	0.70 ± 0.05^a^	1.01 ± 0.18^b^	0.90 ± 0.04^a^	1.55 ± 0.31^c^	1.86 ± 0.15^d^
2,7-Dimethyl-1-octanol	1.36 ± 0.01^a^	1.68 ± 0.32^b^	1.24 ± 0.05^a^	1.68 ± 0.19^b^	1.56 ± 0.12^b^
1-Hexanol, 5-methyl-2-(1-methylethyl)-	2.86 ± 0.49^a^	3.17 ± 0.24^b^	3.26 ± 0.30^b^	3.44 ± 0.26^c^	3.55 ± 0.11^c^
2-Butyloctanol	1.2 ± 0.01^a^	1.96 ± 0.17^b^	2.16 ± 0.01^c^	3.26 ± 0.02^d^	3.40 ± 0.01^d^
1-octen-3-ol	0.06 ± 0.05^a^	0.11 ± 0.09^a^	0.19 ± 0.01^a^	0.27 ± 0.12^a^	0.32 ± 0.12^a^
1-pentanol	0.12 ± 0.12^a^	0.24 ± 0.19^b^	0.19 ± 0.01^a^	0.25 ± 0.05^b^	0.34 ± 0.05^b^
Aldehydes (10)					
2,5-Dimethoxybenzaldehyde	1.31 ± 0.72^a^	1.12 ± 0.09^a^	1.50 ± 0.01^b^	1.57 ± 0.03^b^	1.63 ± 0.87^b^
Nonanal	0.87 ± 0.01^a^	1.45 ± 0.62^b^	1.87 ± 1.73^bc^	2.52 ± 0.23^c^	2.61 ± 0.19^c^
heptanal	1.71 ± 0.22^a^	2.16 ± 0.18^b^	2.05 ± 0.08^ab^	2.36 ± 0.10^b^	2.53 ± 0.34^c^
octanal	1.77 ± 0.48^a^	2.38 ± 0.38^c^	2.17 ± 1.55^b^	2.49 ± 0.20^c^	2.37 ± 0.18^c^
(E)-2-heptenal	1.05 ± 0.22^a^	1.75 ± 0.53^b^	1.25 ± 0.33^a^	1.93 ± 0.39^b^	1.87 ± 0.54^b^
(E, E)-2,4-decadienal	1.05 ± 0.12^a^	1.08 ± 0.41^a^	1.1 ± 0.33^a^	1.51 ± 0.11^b^	1.43 ± 0.25^b^
Propanal	2.57 ± 0.13^a^	2.73 ± 0.42^a^	2.62 ± 0.05^a^	2.91 ± 0.11^b^	2.72 ± 0.15^a^
(E)-2-octenal	0.63 ± 0.25^a^	1.42 ± 0.07^bc^	1.2 ± 0.02^b^	1.53 ± 0.08^c^	1.61 ± 0.08^c^
hexanal	1.17 ± 0.39^a^	1.52 ± 0.33^b^	1.68 ± 0.43^b^	2.02 ± 0.07^c^	1.93 ± 0.21^c^
(E, E)-2,4-heptadienal	0.63 ± 0.43^a^	1.64 ± 0.14b^c^	1.49 ± 0.19^b^	1.79 ± 0.04^c^	1.82 ± 0.51^c^
Acids (3)					
nonanoic acid	0.40 ± 0.03^b^	0.29 ± 0.03^a^	0.24 ± 0.04^a^	0.12 ± 0.07^a^	0.13 ± 0.08^a^
heptanoic acid	0.12 ± 0.07^a^	0.1 ± 0.04^a^	0.76 ± 0.01^b^	0.04 ± 0.02^a^	0.05 ± 0.07a
octanoic acid	0.25 ± 0.01^b^	0.19 ± 0.01^a^	0.19 ± 0.01^a^	0.12 ± 0.01^a^	0.08 ± 0.02^a^
taxanes(13)					
Decane	5.81 ± 1.2^c^	4.66 ± 0.53^b^	4.76 ± 1.02^b^	4.18 ± 1.01^a^	3.91 ± 0.80^a^
Tetratetracontane	3.03 ± 0.26^a^	3.02 ± 0.15^a^	2.92 ± 0.38^a^	2.87 ± 0.39^a^	2.72 ± 0.37^a^
Undecane	13.62 ± 2.16^a^	13.64 ± 1.66^a^	11.79 ± 0.22^b^	12.39 ± 0.16^b^	11.06 ± 1.30^b^
Nonadecane	9.75 ± 1.43^b^	9.74 ± 1.02^b^	10.43 ± 2.08^b^	9.73 ± 2.25^b^	8.77 ± 2.16^a^
Hexadecane	23.09 ± 2.75^b^	18.96 ± 3.10^a^	19.97 ± 1.13^a^	16.30 ± 2.53^a^	16.29 ± 1.40^a^
2,4-Dimethylundecane	5.74 ± 0.31^b^	4.01 ± 4.49^a^	4.34 ± 0.24^a^	4.09 ± 0.23^a^	5.19 ± 0.04^a^
2,4-Dimethylheptane	1.21 ± 0.27^a^	1.27 ± 0.16^a^	1.37 ± 0.06^a^	1.20 ± 0.17^a^	1.35 ± 0.05^a^
3-Ethyl-3-methylheptane	0.61 ± 0.02^a^	0.74 ± 0.09^a^	0.77 ± 0.19^a^	0.65 ± 0.16^a^	0.66 ± 0.07^a^
2,3,5-Trimethylhexane	1.51 ± 0.23^b^	1.41 ± 0.38^b^	1.54 ± 0.25^b^	1.29 ± 0.10^a^	1.37 ± 0.18^a^
Heptadecane	3.34 ± 0.27^b^	3.32 ± 0.21^b^	2.70 ± 0.21^a^	2.91 ± 0.03^a^	3.02 ± 0.08^a^
3,3-Dimethylhexane	1.87 ± 0.17^a^	1.73 ± 0.05^a^	1.74 ± 0.24^a^	1.50 ± 0.07^a^	1.66 ± 0.23^a^
2-Methylundecane	3.95 ± 0.34^a^	3.75 ± 0.11^a^	3.84 ± 0.10^a^	3.35 ± 0.01^a^	3.98 ± 0.11^a^
4-Ethyloctane	1.72 ± 0.05^a^	1.85 ± 0.19^a^	1.80 ± 0.12^a^	1.55 ± 0.01^b^	1.79 ± 0.07^a^

Respectively. Different letters for the same index mean significant difference (*p* < 0.05).

The percentages of alcohols, aldehydes, acids and alkanes in the initial samples were 6.96 %, 12.76 %, 0.77 % and 79.53 %. The lipid oxidation reaction occurring by heating, which produces hydroperoxides, which in turn decompose to produce aldehydes and alcohols as flavor substances, including hexanal, heptanal, octanal, (E)-2-heptenal, nonanal, 1-octen-3-ol and other aroma compounds(Ma et al., 2024).

The aldehydes of the oils increased to 17.13 %, 18.94 %, 20.63 % and 23.84 % with the increase of the melting temperature after four different melting processes. The content of which increased with the oxidation process of the increase in the decomposition of linoleic acid during oxidation indicates that linoleic acid decomposes more at higher carburetion temperatures and represents a higher degree of oxidation(Xi et al., 2024). The same trend was observed for alcohols, which are also major secondary products of fat oxidation(Li et al., 2024).

## 4 Conclusion

In conclusion, the quality of infant formula oil after four different melting processes showed different degrees of changes. Firstly, the quality, oxidative stability and flavor of the oils and fats were best maintained at 50 °C for 29 h, and the quality of the oils and fats treated at 60 °C for 24 h decreased slightly compared with that at 50 °C for 29 h, but most of the time, the difference between the two was relatively small. The difference between 70 °C for 20 h and 80 °C for 18 h compared with 50 °C for 29 h is larger. The two processed oil in the quality, oxidative stability and flavor have a greater degree of decline, indicating that the higher melting temperature on the deterioration of the quality of oil to play a dominant role in the long time at a low temperature of melting is more conducive to the preservation of the quality of raw materials for infant formula oil.

## CRediT authorship contribution statement

**Wen Tu:** Writing – original draft, Validation, Methodology, Investigation, Data curation. **Longyu Wan:** Writing – review & editing, Validation, Formal analysis. **Jiaxin Zhang:** Writing – review & editing, Data curation. **Huabing Wang:** Writing – review & editing, Formal analysis. **Yadong Huang:** Methodology, Data curation. **Xu Wang:** Writing – review & editing, Methodology. **Jian He:** Methodology, Data curation. **Wei Zhang:** Validation, Data curation. **Qianyu Zhao:** Writing – review & editing, Supervision, Project administration. **Feng Zhao:** Project administration, Data curation. **Yujun Jiang:** Supervision, Project administration.

## Declaration of competing interest

The authors declare that they have no known competing financial interests or personal relationships that could have appeared to influence the work reported in this paper.

## Data Availability

Data will be made available on request.
